# Gastric lipoma presenting as a giant bulging mass in an oligosymptomatic patient: a case report

**DOI:** 10.1186/1752-1947-6-317

**Published:** 2012-09-24

**Authors:** Francisco Américo Fernandes Neto, Maria Cristina Furian Ferreira, Luiz Carlos Nascimento Bertoncello, André Amate Neto, Wilson Chubassi de Aveiro, Caroline Agnelli Bento, Gustavo Nardini Cecchino, Marco Antonio Mendes Rocha

**Affiliations:** 1 Department of General Surgery, Hospital Municipal Dr. Mário Gatti, Avenida Prefeito Faria Lima 340, Campinas, São Paulo, 13036-220, Brazil; 2 Hospital e Maternidade Celso Pierro, Avenida John Boyd Dunlop – s/n°, Campinas, São Paulo, 13060-904, Brazil; 3 Pontifícia Universidade Católica de Campinas, Faculty of Medicine, Avenida John Boyd Dunlop – s/n°, Campinas, São Paulo, 13060-904, Brazil

**Keywords:** Neoplasms, Lipoma, Gastrointestinal tract, Stomach, Gastrectomy

## Abstract

**Introduction:**

Lipomas of the gastrointestinal tract are a rare condition. Only 5% are of gastric origin, and this corresponds to 2% to 3% of all benign tumors of the stomach and less than 1% of all gastric neoplasms. It is our purpose to report an unusual presentation of a giant gastric lipoma in an oligosymptomatic patient and highlight the importance of discussing differential diagnosis in this situation. A review of the literature has shown that this is one of the largest gastric lipomas described.

**Case presentation:**

We describe a rare case of a benign gastric tumor with uncommon features in a 63-year-old Caucasian woman. She was admitted with abdominal discomfort, nausea, and upper abdominal fullness after eating. The lesion was suspicious of malignancy because of its dimension and central contrast enhancement on computed tomography. Conventional upper digestive endoscopy revealed a large bulging mass in the gastric posterior wall and three ulcerated areas. In this procedure, a technical limitation due to the location of the mass in the submucosa prevented an adequate biopsy from being obtained. The fragments obtained from the ulcers revealed nothing but necrotic mucosa. Our patient underwent a subtotal gastrectomy and D1 lymphadenectomy with a Roux-en-Y reconstruction. Macroscopic findings revealed a 12 × 8 × 6cm mass with a volume of 576cm^3^, and the histological pattern demonstrated well-differentiated mature adipose tissue surrounded by a fibrous capsule, confirming the diagnosis of gastric submucosal lipoma.

**Conclusions:**

Gastric lipoma is a rare benign disease that eventually simulates a malignant tumor.

## Introduction

Lipomas of the gastrointestinal tract are rare: only 5% are of gastric origin. They account for 2% to 3% of all benign tumors of the stomach and less than 1% of all gastric neoplasms [1-3]. Generally unusual and slow-growing, they are localized in the submucosa in 90% to 95% of cases and in the serosa in 5% to 10% of cases. However, regardless of the layer involved, 75% affect the antrum [1,4]. We describe an unusual presentation of a giant gastric lipoma and highlight the importance of discussing its differential diagnosis. A review of the literature has shown that this is one of the largest gastric lipomas described.

## Case presentation

**Figure 1:**
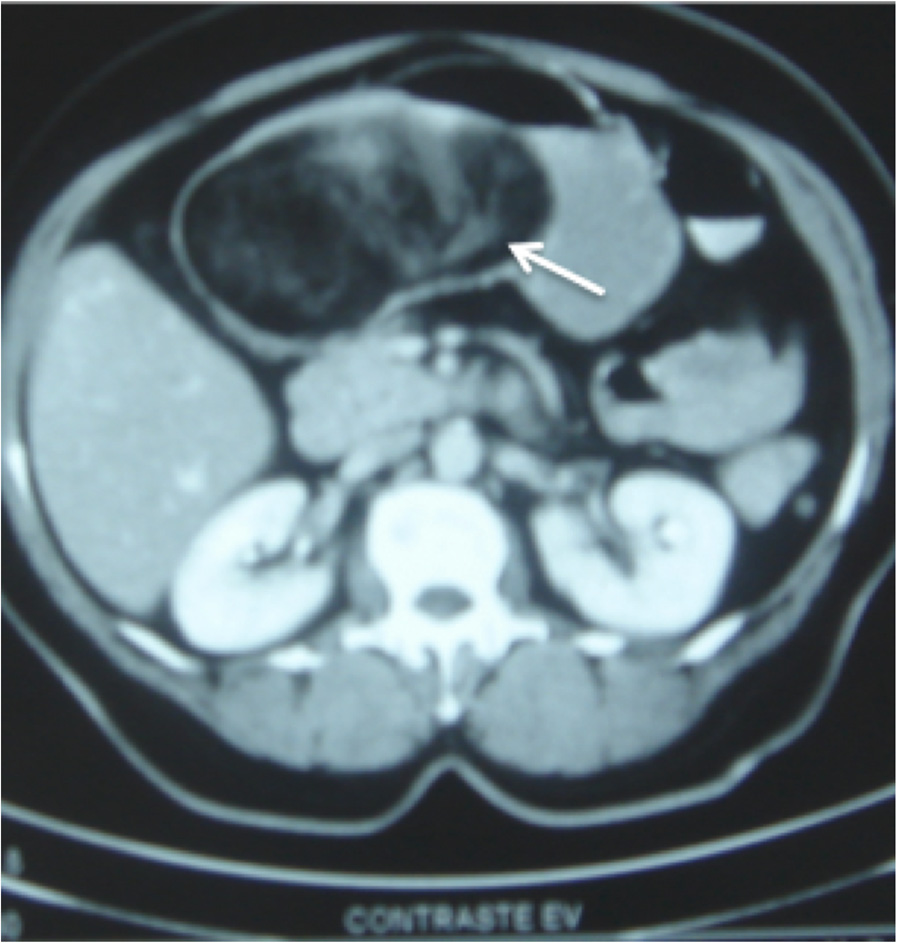
A computed tomography image before surgical exploration shows a large homogeneous mass with fat tissue density and central contrast captation with trabecular architecture (white arrow).

A 63-year-old Caucasian woman complaining of insidious upper abdominal pain (unrelated to food ingestion) and nausea was admitted after an episode of intense acute abdominal pain. She denied having hematemesis or melena and had a history of untreated dyslipidemia. Her dietary habits included acid fruits, soft drinks, beer, chocolate, and high-fat foods. She was a smoker of 10 or more cigarettes per day and had no family history of cancer. She had systemic arterial hypertension that was being treated with 500mg of methyldopa per day. The results of a physical examination were normal except for a palpable and movable upper abdominal mass. The results of all laboratory tests were normal, and our patient did not have anemia. An abdominal ultrasound was promptly performed, and a large echoic mass compatible with an expansive lesion was found in the gastric antrum. Conventional endoscopy detected a large bulging mass in the posterior gastric wall and three ulcerated areas in the gastric mucosa, but none of them showed signs of recent bleeding (Forrest III). Owing to technical limitations, the procedure did not result in an adequate biopsy of the mass in the submucosa. The fragments acquired from the ulcers revealed nothing but necrotic mucosa. Abdominal contrasted computed tomography revealed a well-defined homogeneous oval mass that was located within the posterior gastric wall and that compressed the second portion of the duodenum. The lesion had fat tissue density and a suspicious central contrast enhancement with trabecular architecture but was noninvasive with respect to the peripheral layers (Figure 1).

**Figure 2:**
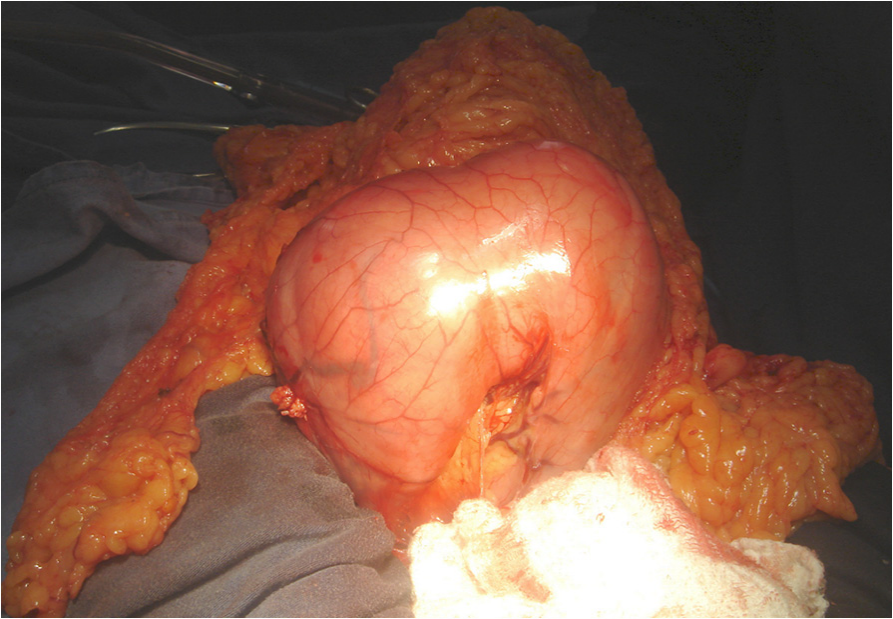
Intra-operative aspect of the massive gastric lipoma surrounded by a fibrous capsule and no signs of lymphadenopathy or local metastasis.

For this reason, as well as the tomographic dimensions (size of 10 × 6 × 11cm and volume of 660cm^3^), malignancy was suspected. Our patient then underwent an open laparotomy for abdominal exploration. During surgery, unexpected benign aspects of the mass, such as the envelopment by a fibrous capsule and no signs of lymphadenopathy or local metastasis, surprised the team (Figure 2). Multiple septated cysts in the right ovary and multiple simple cysts in the left ovary were also found, and although the freezing biopsy failed to demonstrate tumor cells, a bilateral oophorectomy was done. In regard to the gastric mass, subtotal gastrectomy and D1 lymphadenectomy with a Roux-en-Y reconstruction were performed. The post-operative period was uneventful, and our patient was discharged by the seventh day.

**Figure 3:**
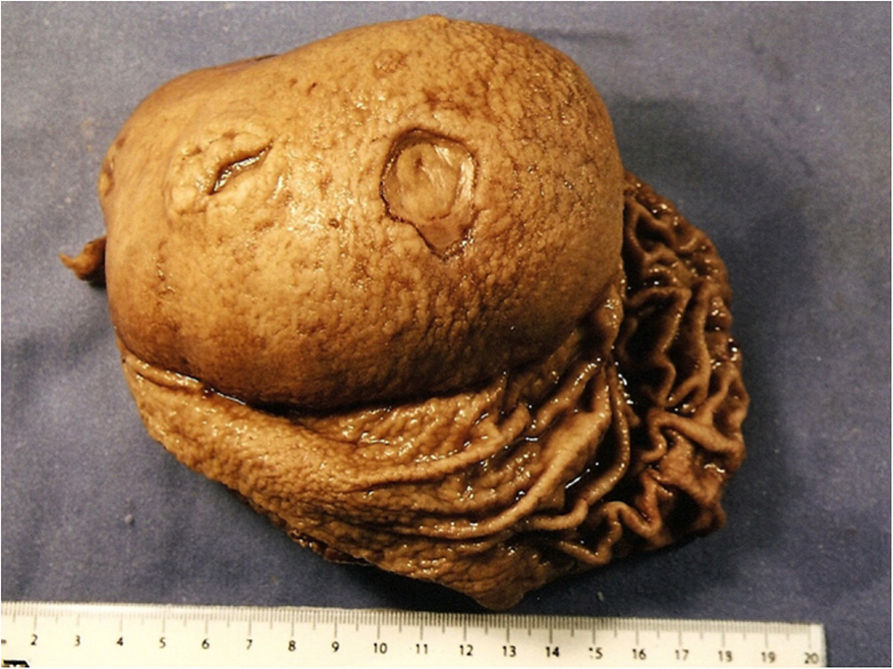
**A surgical specimen of open stomach through the lesser curve discloses a large mass that is located over the posterior gastric wall and covered by mucosa.** Two ulcerated areas are also disclosed.

**Figure 4:**
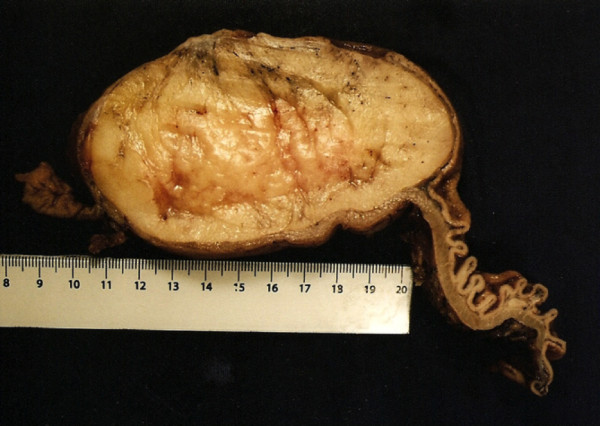
A cut surface of the surgical specimen reveals a well-defined homogeneous aspect of the lipoma and the cleavage plane.

**Figure 5:**
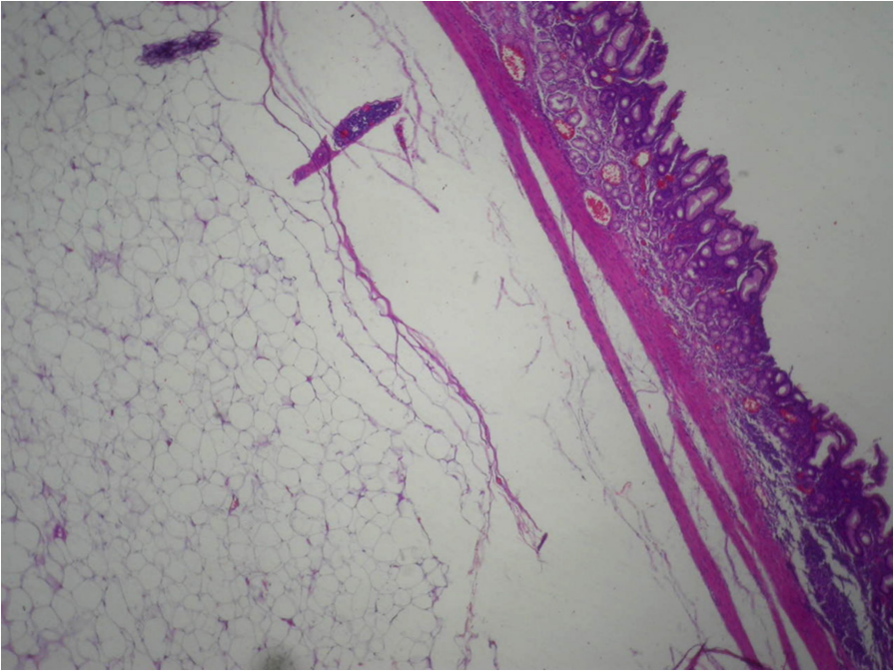
**A histological study of the gastric layers exhibits the noninvasive submucosal lesion.** Stain: hematoxylin and eosin; magnification: ×200.

**Figure 6:**
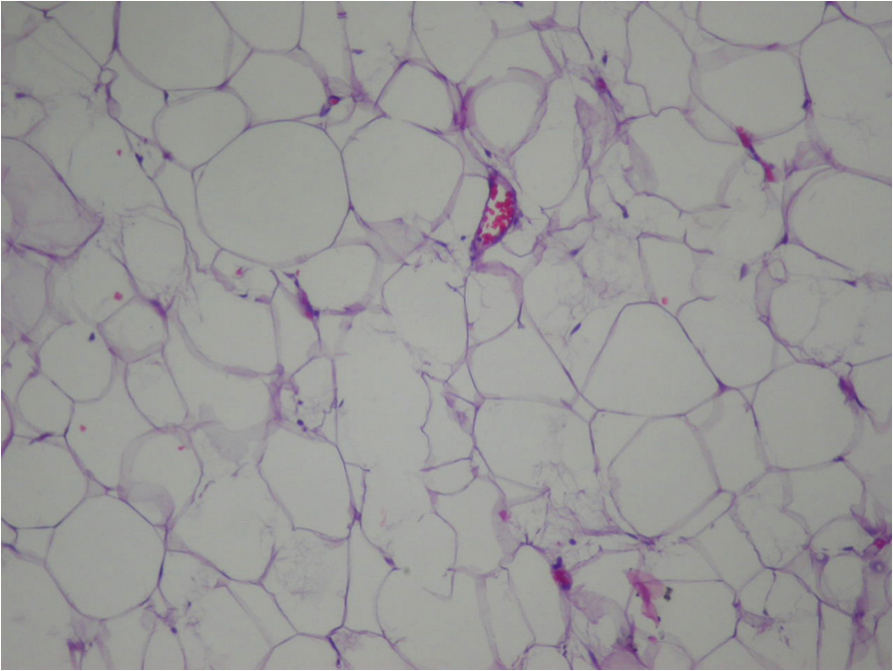
**Pathologic findings of the lesion reveal well-differentiated mature adipocytes.** Stain: hematoxylin and eosin; magnification: ×400.

Macroscopic findings included a well-defined homogeneous yellowish lesion that was located in the submucosa and that comprised the entire gastric posterior wall and three ulcerated zones of 0.5, 1.0, and 1.4cm in diameter (Figures 3 and 4). Despite the dimension indicated by computed tomography, an actual size of 12 × 8 × 6cm (576cm^3^) was measured. Microscopy revealed a histological pattern of well-differentiated noninvasive mature adipose tissue surrounded by a fibrous capsule (Figures 5 and 6). The findings confirmed the benign intra-operative aspect of the lesion and the diagnosis of gastric submucosal lipoma. The greater omentum was free of tumor cells.

## Discussion

Lipomas are benign soft tissue tumors that are composed of mature lipocytes and circumscribed by a fibrous capsule. Macroscopically, lipomas are formed by yellowish fat tissue of various sizes [4,5]. Even though the etiology remains unknown, studies suggest that lipomas might be related to an embryological sequester of adipocytes or even be due to the natural process of aging [4,5].

In 1955, Weinberg and Feldman [6] reported 135 cases of lipoma in the gastrointestinal tract through a meta-analysis of 60,000 autopsies [7]. In 1963, a study of 4000 surgical resections of benign tumors of the gastrointestinal tract by Mayo *et al.*[8] revealed that the incidence of lipoma was about 4%, which was distributed as follows: the esophagus (1.6%), followed by the stomach (3.2%), duodenum/small intestine (31.2%), and, most frequently, the colon (64%) [9].

Gastric lipomas prevail between the fifth and seventh decades of life, are found mainly in women, and usually are small and asymptomatic and are detected incidentally [3,4,10]. They are rarely large, intramuscular, or poorly circumscribed but rather are usually smooth, mobile, and painless masses [1,4,5,10]. Turkington [1], in 1965, reviewed 157 cases in the literature and demonstrated that lipomas may arise from every part of the stomach but less frequently from the cardia and pylorus.

The occurrence of symptoms depends on the size and location of the tumor. Lesions of less than 2.0cm are usually asymptomatic. In patients with larger lesions, the most common symptoms are hemorrhage, abdominal pain, pyloric obstruction, and dyspepsia. Additional symptoms may include diarrhea, constipation, and intussusception [1,4,10,11]. Gastrointestinal bleeding is typically chronic and minimal and is able to cause anemia [10]. Thus, it is noteworthy that, in spite of the volume and extension of the mass, our patient was oligosymptomatic, presented no bleeding or anemia, and reported only upper abdominal discomfort and fullness.

Lately, new imaging techniques such as conventional endoscopy and endoscopic sonography have become important tools for investigating gastric lipomas. Conventional endoscopy reveals lipomas as smooth, oval or round, yellowish, solitary, protruding masses that are covered by mucosa and that may present ulcerated areas. Classic endoscopic features are highly suggestive, such as the “tenting sign” (overlying mucosa may be observed), the “cushion sign” (the smooth consistency of the lesion may be flattened and restored), and the “naked fat sign” (fat extrusion is detected after biopsy) [4,10-13].

Biopsy through conventional endoscopy reveals only a typical mucosa and thus is not able to confirm diagnostic suspicions [12,13]. In the case described, only conventional endoscopy was available. Therefore, the biopsy fragments from the ulcers could not securely indicate whether the underlying submucosal mass was benign, an indication that could have led to a less aggressive treatment choice. Recent literature points out the possibility of performing methods other than the lift-and-cut biopsy technique for submucosal sampling, such as echo-guided punction and diathermal loop biopsy [14].

The use of endoscopic sonography is useful for identifying the tumor’s primary layer, which is best for describing the lesion’s morphology, and reveals possible invasion of the lymph nodes and peripheral layers. Typically, lipomas are visualized as hyperechoic homogeneous lesions that have regular margins and that arise from the third layer [12-14]. The isolated use of such technology has a low accuracy in diagnosing subepithelial lesions and must be complemented by endoluminal resection technologies for histological confirmation [14]. Because endoscopic sonography was not available for resolving the reported case, the primary suggested diagnosis was a possible malignant tumor.

The best noninvasive exam for large gastrointestinal lipomas is abdominal computed tomography, which is sufficient yet not definitive for diagnosis [5]. Imaging findings include a well-delimited homogeneous gastric mass with a density of between −70 and −120 Hounsfield units. Other characteristics are ulceration of the mucosa correlated with the presence of a fibrovascular septum and linear margin with soft tissue density. Such aspects exclude the malignant hypothesis of liposarcoma, thus discarding the need for a biopsy [5,10,11]. In this case, because computed tomography was inconclusive and the biopsies from the conventional endoscopy were unhelpful, the hypothesis of malignancy could not be excluded. Therefore, the team decided to perform a gastrectomy, which would be unnecessary in other circumstances.

Incidental lipomas should not be treated, as there are no reports of malignant degeneration [1,12,13]. Lesions of less than 6.0cm in diameter or with endoluminal or extraluminal protrusion should undergo laparoscopic resection [11,13]. Surgical excision is indicated because of symptoms, imminent life-threatening risk, and the impossibility of excluding malignancy [11-13]. Endoscopic resection has become important since frequent uneventful surgical and post-operative reports were published [3,13]. The most important differential diagnosis for gastric lipoma aside from liposarcoma is a gastrointestinal tract soft tissue tumor such as a gastrointestinal stromal tumor, leiomyoma, fibroma, and their malignant variables. Occasionally, gastric lipomas may also have to be distinguished from other types of intramural tumors, such as the neurilemoma, adenomyoma, Brunner’s gland adenoma, and heterotopic pancreas [15].

## Conclusions

Gastric lipoma is a rare benign disease that eventually simulates a malignant tumor. This case is presented on account of its rarity and the surprisingly few symptoms reported by the patient given the dimensions and tomographic signs of malignancy. Thus, it is best advised to thoroughly discuss the differential diagnosis for a gastric submucosal mass in order to plan for the best treatment.

## Consent

Written informed consent was obtained from the patient for publication of this case report and accompanying images. A copy of the written consent is available for review by the Editor-in-Chief of this journal.

## Competing interests

The authors declare that they have no competing interests.

## Authors’ contributions

FAFN was the chief surgeon and provided the medical documents for the reported case. MCFF provided the histological examination of the gastric tumor, supervised the study, and performed critical revision of the manuscript for important intellectual content. LCNB provided technical support and performed critical revision of the manuscript for important intellectual content. AAN and WCA revised the patient’s medical records and obtained informed consent. GNC and CAB performed acquisition, analysis, and interpretation of data, drafted the manuscript, and translated the final version. MAMR performed acquisition, analysis, and interpretation of data and drafted the manuscript. All authors read and approved the final manuscript.

## References

[B1] Turkington RW Gastric lipoma: report of a case and review of the literature Am J Dig Dis 1965 10 719 726 10.1007/BF02236072 14316760

[B2] Athanazio DA Motta MP Motta A Lanat L Athanazio PRF A rare case of submucosa lipoma mimicking a malignantgastric tumor J Port Gastrenterol 2008 15 37 38

[B3] Krasniqi AS Hoxha FT Bicaj BX Hashani SI Hasimja SM Kelmendi SM Gashi-Luci LH Symptomatic subserosal gastric lipoma successfully treated with enucleation World J Gastroenterol 2008 14 5930 5932 10.3748/wjg.14.5930 18855998 PMC2751909

[B4] Saltzman JR Carr-Locke DL Fink SA Lipoma case report MedGenMed 2005 7 16 16369321 PMC1681394

[B5] Thomson WM Kende AI Levy AD Imaging chacacteristics of gastric lipomas in 16 adult and pediatric patients Am J Roentgenol 2003 181 981 985 10.2214/ajr.181.4.1810981 14500213

[B6] Weinberg T Feldman M Lipomas of the gastrointestinal tract Am J Clin Pathol 1955 25 272 281 14361323 10.1093/ajcp/25.3.272

[B7] Zhang X Ouyang J Kim Y Large ulcerated cecal lipoma mimicking malignancy World J Gastrointest Oncol 2010 2 304 306 10.4251/wjgo.v2.i7.304 21160661 PMC2999136

[B8] Mayo CW Pagtalunan RJG Brown DJ Lipoma of the alimentary tract Surgery 1963 53 598 603 13934160

[B9] Yamane T Uchiyama K Furuya T Ishii T Omura N Nakano M Fukamachi S Suwa T Okusa T A case of lipoma of the stomach prolapsing into the duodenal bulb and causing a duodenal ulcer Nihon Shokakibyo Gakkai Zasshi 2009 106 1643 1649 19893295

[B10] Taylor AJ Stewart ET Dodds WJ Gastrointestinal lipomas: a radiologic and pathologic review Am J Roentgenol 1990 155 1205 1210 10.2214/ajr.155.6.2122666 2122666

[B11] Singh R Bawa AS Lipoma of the stomach Indian J Surg 2004 66 177 179

[B12] Krinsky M Binmoeller K Endoscopic ultrasound for the characterization of subepithelial lesions of the upper gastrointestinal tract http://www.uptodate.com/contents/topic.do?topicKey=GAST/2666

[B13] Sadio A Peixoto P Castanheira A Cancela E Ministro P Casimiro C Silva A Gastric lipoma – an unusual cause of upper gastrointestinal bleeding Rev Esp Enferm Dig 2010 102 398 400 20575608 10.4321/s1130-01082010000600016

[B14] Karaca C Turner BG Cizginer S Forcione D Brugge W Accuracy of EUS in the evaluation os small gastric subepithelial lesions Gastrointest Endosc 2010 71 722 727 10.1016/j.gie.2009.10.019 20171632

[B15] Treska V Pesek M Kreuzberg B Chudácek Z Ludvíková M Topolcan O Gastric lipoma presenting as upper gastrointestinal obstruction J Gastroenterol 1998 33 716 719 10.1007/s005350050160 9773937

